# Enhanced Recovery After Surgery Protocols in Obese Gynecological Oncology Patients: A Single-Center Experience

**DOI:** 10.7759/cureus.40453

**Published:** 2023-06-15

**Authors:** Anastasios Pandraklakis, Dimitrios Haidopoulos, Theodoros Lappas, Emmanouil Stamatakis, Maria D Oikonomou, Dimitrios Valsamidis, Alexandros Rodolakis, Nikolaos Thomakos

**Affiliations:** 1 Division of Gynaecologic Oncology, 1st Department of Obstetrics and Gynaecology, Alexandra Hospital, National and Kapodistrian University of Athens School of Medicine, Athens, GRC; 2 Department of Anaesthesiology, Alexandra General Hospital, Athens, GRC; 3 The Fertility Centre, Chelsea and Westminster Hospital NHS Foundation Trust, London, GBR

**Keywords:** gynaecology, obesity, eras, enhanced recovery after surgery, gynaecological oncology

## Abstract

Objective

The aim of this study is to present our experience and evaluate the safety and outcomes of the implementation of Enhanced Recovery After Surgery (ERAS) protocols in obese patients who underwent surgery for suspected or confirmed gynecological malignancies.

Method

From January 2020 to September 2021, 217 patients underwent laparotomy for a confirmed or suspected gynecological malignancy following a 19-element ERAS pathway. The patients were divided into two groups: obese (BMI ≥ 30 kg/m^2^, n = 104) and non-obese (BMI < 30, n = 113). Both groups were treated with a 19-element ERAS protocol.

Results

After dividing the 217 patients into two groups, significantly more comorbidities were observed in the obese group (diabetes mellitus: 23% vs. 8%, p = 0.004; ASA score grade 3: 25.0% vs. 6.2%, p < 0.001), as well as higher rates of endometrial cancer (51.9% vs. 17.7%, p < 0.001) compared to the non-obese group. The overall ERAS compliance rates when matched element by element were similar. Postoperatively, complication rates of all grades were significantly higher in the obese group (46.1% vs. 27.4%, p < 0.001) without differences in the length of stay, readmission, and reoperation rates.

Conclusion

In this retrospective study, we showed that obese gynecological oncology patients can be safely managed with ERAS protocols perioperatively while potentially minimizing the adverse outcomes in these otherwise high-risk patients.

## Introduction

Enhanced Recovery After Surgery (ERAS) protocols were introduced in the last decades as an evidence-based multi-elemental and standardized perioperative package of care that provides improved perioperative outcomes, but how can this be achieved? The hypothesis of the effectiveness of these modern protocols is that when evidence-based perioperative interventions are combined together, they can maintain the surgically induced stress at low levels and accelerate postoperative recovery [1]. Practically, when these protocols are successfully applied, they can reduce postoperative complications by 32%, the length of hospital stay (LOS) by 1.6 days, and the readmission rates by 20% [2]. These protocols were introduced in the 1990s by colorectal surgeons and the first guidelines for the application of ERAS protocols in gynecological oncology were published by the ERAS® Society in 2016 [3] and were recently updated in 2023 [4].

Obesity is a global issue with increasing prevalence, especially in populations with low socioeconomic status [5]. It is generally known that this group of patients is at high risk of worse postoperative outcomes [6]. It is worth mentioning that almost half of the gynecological oncology patients who require abdominal surgery are obese, which poses an extra difficulty in their treatments. ERAS protocols have shown evidence of decreasing morbidity rates in obese and non-obese patients in multiple specialties. The aim of this study is to assess the safety of ERAS protocols and provide further evidence, comparing the compliance to the protocol and the perioperative outcomes between obese and non-obese patients who underwent open abdominal surgery at our gynecological oncology department.

## Materials and methods

This study is a single-center retrospective cohort study of patients who underwent laparotomy for a suspected or diagnosed gynecological malignancy and who were perioperatively managed with a 19-element ERAS protocol between January 2020 and September 2021 at the Division of Gynecological Oncology, which is a part of the Obstetrics and Gynecology Department of Alexandra University Hospital in Athens, Greece. All gynecological oncology patients undergoing an elective open (laparotomy) approach were considered eligible. All patients were included without any inclusion or exclusion criteria, as recommended by the ERAS® Society.

The preoperative assessment included signing a written consent to undergo surgery. To include the patients in their care, training and informational leaflets on the scope of the procedure and the perioperative period were given to each patient. Nutritional recommendations were also provided for a high-protein diet. Up until the evening before the procedure, a light, solid diet was undertaken. All patients were managed according to a 19-element ERAS perioperative protocol, as described in Table 1.

**Table 1:** ERAS protocol ERAS: Enhanced Recovery After Surgery; PONV: postoperative nausea and vomiting; NGT: nasogastric tube; POD: postoperative day.

Protocol element	Specific
Preadmission counseling	Information, education, nutritional support, and recommendation to stop smoking
No bowel preparation	Rectal enema given the night before the surgery
Oral carbohydrate loading	No fasting, carbohydrate loading clear liquid 6 and 3 hours before surgery
Avoid long-acting	Avoidance of long-acting sedatives starting from the day before surgery
Thrombosis prophylaxis	A prophylactic dose of low molecular weight heparin the night before and 28 days following the surgical procedure
Antibiotic prophylaxis	Intravenous antibiotics 60’ before the start of the operation
PONV prophylaxis	Multimodal approach: dexamethasone and ondansetron at the induction of anesthesia and droperidol at the end of the operation
Avoidance of systemic opioids	Avoidance of intravenous, oral, or intramuscular opioid use
Avoid epidural analgesia	Patient’s decision after discussion with the anesthetic team
Active warming	Air heating cover, warming mattress
No NGT	Avoidance of nasogastric tube or removal of the NGT at the end of the procedure
No drains	Avoidance of abdominal drains
Urinary catheter removal on POD1	Urinary catheter removal on POD1
Stimulation of gut motility	Lactulose, chewing gum, and coffee on POD1
Prompt termination of intravenous fluids	Termination of intravenous fluids on POD1
Early feeding	Oral liquid or light diet on the day of operation (300 kcal/day) and light or normal diet the day after (600 kcal/day)
Early mobilization	Active mobilization on the day of the operation (POD0)
30-day follow up	Outpatient follow-up within 30 days postoperatively

Following surgery, each patient was sent to the high dependency unit (HDU) and then to the ward on postoperative day (POD) one. On POD0, active mobilization and a light solid diet were initiated with the intention of stopping IV fluids on POD1. Patients fulfilled the requirements for discharge when they could mobilize freely, tolerated oral nutrition, and used oral analgesics to treat postoperative pain.

We collected demographic and clinical information, such as age and body mass index (BMI) at the time of surgery, American Society of Anesthesiologists (ASA) score, history of diabetes mellitus (DM), prior chemotherapy or prior radiotherapy, tumor type, surgical complexity score, LOS, 30-day readmission, 30-day reoperation, 30-day perioperative complications, overall compliance with the ERAS program, and compliance of each individual ERAS element. We also analyzed the total intravenous postoperative opioid use. To avoid any bias in this analysis, we included only the patients who were managed without epidural analgesia. We classified perioperative complications using the Clavien-Dindo classification system [7] and we characterized surgical complexity as described by Aletti et al. [8].

We divided patients into non-obese (normal weight and overweight, BMI = 18.5-29.9 kg/m2) and obese (BMI ≥ 30.0 kg/m2) groups based on their BMI at the time of surgery according to the World Health Organization (WHO) [9] and we compared all the collected data between these two groups. The study's main findings included readmission rates within 30 days following surgery, LOS, and complication rates. Compliance with the ERAS protocol, pain levels on POD0 and POD1 (Visual Analog Scale, VAS) [10], time to flatus, and nausea on the day of the procedure were considered secondary outcomes. VAS score and nausea were assessed by the nursing staff and the physiotherapists and time to flatus was reported by the patients.

Patient demographics, clinical characteristics, and outcomes were presented with descriptive statistics, and data were compared using the Fisher’s exact test or chi-squared test for categorical variables and the Kruskal-Wallis test for continuous variables. Statistical significance was set at p < 0.05, and analyses were conducted using the Jamovi statistical software (version 2.3.21.0). The structure of the study was based on the recommendations of the Strengthening the Reporting of Observational Studies in Epidemiology (STROBE) statement [11] and on the Reporting on ERAS Compliance, Outcomes, and Elements Research (RECOvER) checklist recommendations by the ERAS® Society [12].

## Results

A total of 217 patients were enrolled in our study: 113 non-obese patients and 104 obese patients. Patient demographics (Table 2), clinical and surgical characteristics (Table 3), and postoperative outcomes (Table 4) are presented below. A statistically significant difference was observed in patients’ comorbidities. Obese patients had significantly higher rates of DM (23% vs. 8%, p = 0.004), lower ASA 1 (2.9% vs. 73.5%, p < 0.001), and higher ASA 3 scores (25.0% vs. 6.2%, p < 0.001). Furthermore, the obese group had significantly higher numbers of endometrial cancer patients (51.9% vs. 17.7%, p < 0.001) and a non-significantly lower number of ovarian cancer cases (28.8% vs. 48.7%, p = 0.013). No significant differences were observed between the two groups in mean age, smoking, preoperative chemotherapy and radiotherapy, surgical complexity, and other tumor types.

**Table 2:** Patient demographics ASA: American Society of Anesthesiologists physical status.

	Total sample (n = 217)	Obesity		p-value
		No (n = 113; 52.1%)	Yes (n = 104; 47.9%)	
Age, mean (SD)	59.1 (13)	56.95 (13.2)	61.37 (12.3)	0.012
ΒΜΙ, mean (SD)	30.2 (5.8)	25.4 (1.95)	34.2 (5.89)	N/A
Smoking, n (%)	70 (32.3)	33 (29.2)	37 (35.5)	0.316
Diabetes, n (%)	34 (15.7)	10 (8.8)	24 (23)	0.004
Chemotherapy in the past, n (%)	23 (10.6)	9 (8.1)	14 (12.7)	0.189
Radiotherapy in the past, n (%)	3 (1.4)	1 (1.0)	2 (1.7)	0.513
ASA score, n (%)				
1	86 (39.6)	83 (73.5)	3 (2.9)	<0.001
2	97 (44.7)	22 (19.5)	75 (72.1)	0.020
3	33 (15.2)	7 (6.2)	26 (25.0)	<0.001
4	1 (0.5)	1 (0.8)	0 (0.0)	0.584

**Table 3:** Clinical and surgical characteristics

		Total sample (n = 217)	Obesity		p-value
			No (n =113; 52.1%)	Yes (n = 104; 47.9%)	
Diagnosis, n (%)					
	Benign	32 (14.8)	20 (17.7)	12 (11.5)	0.100
	Borderline ovarian tumor	1 (0.5)	1 (0.9)	0 (0.0)	0.584
	Cervical cancer	15 (6.9)	11 (9.7)	4 (3.9)	0.236
	Endometrial cancer	74 (34.1)	20 (17.7)	54 (51.9)	<0.001
	Ovarian cancer	85 (39.1)	55 (48.7)	30 (28.8)	0.013
	Other	2 (0.9)	1 (0.9)	1 (1.0)	0.594
	Uterine cancer (sarcoma)	8 (3.7)	5 (4.4)	3 (2.9)	0.559
Surgical complexity					
Aletti score, mean (SD)		3.01 (1.49)	3.04 (1.68)	2.99 (1.27)	0.825
Aletti score, n (%)					
	<4	146 (67.2)	74 (65.4)	72 (69.2)	0.514
	>=4	71 (32.8)	37 (32.7)	32 (30.7)	

**Table 4:** Postoperative outcomes VAS: visual analog scale; IQR: interquartile range; SD: standard deviation; POD: postoperative day.

	Total sample (n = 217)	Obesity				p-value
		No (n = 113; 52.1%)		Yes (n = 104; 47.9%)		
VAS POD0, median (IQR)	3 (3-4)	4 (3-4)		3 (3-4)		0.574
VAS POD1, median (IQR)	3 (2-3)	3 (2-3)		2 (2-4)		0.133
Flatus (days), mean (SD)	1.89 (0.73)	1.84 (0.69)		1.94 (0.73)		0.344
Nausea on POD0	51 (23.5)	29 (25.7)		22 (21.2)		0.434
Total intravenous postoperative opioid dose, (morphine equivalent) (mg) (mean) * mean (SD)	53.85 (19.95)	55.12 (19,65)		52.8 (20,4)		0.220
Length of stay (days), median (IQR)	4 (3-5)	4 (3-5)		4 (3-5)		0.528
Readmission, n (%)	12 (5.5)	8 (7)		4 (3.8)		0.127
Reoperation, n (%)	2 (0.9)		0 (0)		2 (1.9)	0.139
Complications, n (%)	79 (36.4)		31 (27.4)		48 (46.1)	<0.001
Clavien-Dindo score, n (%)						
I	54 (24,8)		18 (15.9)		36 (34.6)	0.016
II	20 (9.2)		10 (8.8)		10 (9.6)	0.410
III	5 (2.3)		3 (2.6)		2 (1.9)	0.432
IV	0 (0.0)		0 (0.0)		0 (0.0)	1

In terms of compliance with the ERAS protocol, both obese and non-obese groups had a similar mean overall compliance (83.3% obese group vs. 83.9% non-obese group). Regarding each ERAS element separately, no statistically significant differences were observed between the two groups (Table 5).

**Table 5:** Compliance with ERAS elements ERAS: Enhanced Recovery After Surgery; PONV: postoperative nausea and vomiting; NGT: nasogastric tube.

	Total sample (Ν = 217)	Obesity		p-value
		No (n = 113; 52.1%)	Yes (n = 104; 47.9%)	
	n (%)	n (%)	n (%)	
Pre-op				
Pre-admission education	217 (100.0)	113 (100.0)	104 (100.0)	1
No bowel preparation	215 (99.1)	112 (99.1)	103 (99.0)	0.953
Oral carbohydrates	212 (97.7)	109 (96.5)	103 (99.0)	0.208
Avoid long-active sedatives	214 (98.6)	111 (98.2)	103 (99.0)	0.612
Thrombosis prophylaxis	217 (100.0)	113 (100.0)	104 (100.0)	1
Antibiotic prophylaxis	217 (100.0)	113 (100.0)	104 (100.0)	1
PONV prophylaxis	217 (100.0)	113 (100.0)	104 (100.0)	1
Peri-op				
Avoid epidural	99 (45.6)	45 (39.8)	54 (51.9)	0.074
Avoid systemic opioids	98 (45.1)	55 (48.6)	43 (41.3)	0.406
Air heating body cover	217 (100.0)	113 (100.0)	104 (100.0)	1
No NGT	214 (98.6)	112 (99.1)	102 (98.1)	0.515
No drains	32 (14.7)	22 (19.5)	10 (9.6)	0.041
Post-op				
Prompt termination of urinary drainage (POD1)	123 (56.7)	71 (62.8)	52 (50)	0.057
Stimulation of gut motility	201 (92.6)	104 (92.0)	97 (93.3)	0.730
Prompt termination of intravenous fluids (POD1)	171 (78,8)	85 (75.2)	86 (82.7)	0.180
Oral nutrition on POD0 > 300 kcal	189 (87.1)	100 (88.5)	89 (85.6)	0.524
Oral nutrition on POD1 > is 600 kcal	212 (97.7)	111 (98.2)	101 (97.1)	0.587
Mobilization POD0	168 (77.4)	88 (77.9)	80 (76.9)	0.868
30-day follow up	217 (100.0)	113 (100.0)	104 (100.0)	1
ERAS compliance (%)	83.6	83.9	83.3	

Postoperatively, the obese group had significantly higher complication rates of any grade (46.1% vs. 27.4%, p < 0.001), without significant differences when complications were matched according to the Clavien-Dindo classification. However, patients in the obese group had almost double chances of grade I postoperative complication (34.6% vs. 15.9%, p = 0.016). Both groups had similar LOS, readmission and reoperation rates, postoperative pain levels on POD0 and POD1, time to flatus, nausea on POD0, and the total amount of opioids postoperatively. Hospitalization due to readmission has been included in the analysis apart from one patient in the obese group who had a prolonged length of stay (25 days). Eight patients in the non-obese group were readmitted: two for postoperative ileus; four for pyrexia, surgical site infection, or urinary tract infection, who required intravenous antibiotics; and two for postoperative collections, who required computed tomography-guided drainage. Four patients in the obese group were readmitted: one for surgical site infection (necrosis), who required reoperation; two for pyrexia or sepsis, who required intravenous antibiotics (n = 2); and one for postoperative ileus. One more patient in the obese group required reoperation due to an anastomotic leak.

## Discussion

In our institutional retrospective cohort study, we showed that ERAS protocols can be safely and effectively implemented in obese patients with similar compliance rates to those of non-obese patients. Although obese patients are at higher risk of developing postoperative complications of any grade compared to the non-obese group, this is predominantly due to an increased risk of developing grade I postoperative complications, as there is no significant effect on the postoperative outcomes such as the LOS, readmission, and reoperation rates.

Obesity is an issue rising worldwide and has nearly tripled since 1975 [13]. High-BMI patients are always a challenge not only for the surgeon but also for the anesthetist as the excess of adipose tissue can increase the risk of perioperative anesthetic [14] and surgical complications while it can also increase surgical difficulty, intraoperative surgical time, and the risk of bleeding. Obesity is also related to significant comorbidities such as type 2 DM and cardiovascular, pulmonary, and other metabolic diseases [15], with a higher incidence of multiple malignancies, including gynecological cancers such as endometrial, ovarian, and breast cancer [16]. This study observed similar findings to the above, as patients in the obese group had significantly increased DM rates and ASA scores.

According to the WHO classification system for obesity, there are three subgroups with increasing BMI: class I (BMI = 30-35 kg/m2, class II (BMI = 35-40 kg/m2), and class III (BMI > 40 kg/m2) [9]. Choi et al. in a retrospective cohort study including 15,682 emergency and elective colorectal surgery patients showed that class III (morbid) obesity is a significant risk factor for postoperative complications compared to class I and normal BMI patients [17]. Our study enrolled mostly class I patients (n = 78, 75%), and therefore, this hypothesis could not be assessed without significant bias.

The safety of the implementation of ERAS protocols in this group of patients has also been assessed by Harrison et al., presenting similar outcomes to our study [18]. In this study, the authors showed that obese patients had increased rates of grade I and II complications (17.8% vs. 4.9%, p < 0.001) and similar compliance to ERAS elements, which are also consistent with our findings. However, Iranmanesh et al. did not report significant differences in the postoperative outcomes between obese and non-obese colorectal patients treated with an ERAS protocol, with the only difference being the time of return to normal bowel function (obese = 2.38 days vs. non-obese = 1.98 days, p = 0.03). These findings raise the question, “Could potentially the benefit of an ERAS program eliminate the morbidity gap between obese and non-obese patients?” On the contrary, Mullen et al. presented an “obesity paradox” in two large cohort studies, arguing about the obesity-related risks while showing that obesity was not associated with increased risks of morbidity and mortality [19,20]. In any case, the ERAS Society has made significant progress in creating prehabilitation programs including lifestyle modifications (exercise programs [21,22], nutritional recommendations [23], psychological support [24], and medical treatment of obesity-related dysglycemia [25]). However, there are no standardized protocols and evidence-based recommendations to support a well-structured prehabilitation program for these high-risk patients. Furthermore, one should take into consideration that a further delay to apply a prehabilitation program could potentially lead to disease progression and worse oncologic outcomes. However, active prehabilitation protocols [26] without postponing the surgical treatment could be a beneficial option during the recently increasing delays in cancer treatments in the COVID-19 era [27,28].

The strength of this study is based on the fact that all primary operations were performed in a tertiary gynecological cancer center, a referral center for high-BMI patients, by three gynecological oncology surgeons, and all the anesthetic procedures were performed by three anesthetists experienced in perioperative and postoperative ERAS protocols. Furthermore, all patients were actively mobilized by experienced ERAS specialist staff. The basic limitation was the retrospective design of the study and the fact that it was conducted in a single institution. Some other limitations that must be highlighted are that the current study may not have been able to detect statistically significant relationships due to the small sample size and the few occurrences, age, and comorbidity differences between the groups. Another limitation is that high BMI patients could not be further sub-categorized into obese (BMI = 30-34.9 kg/m2), extremely obese (BMI = 35-39.9 kg/m2), and morbidly obese (BMI > 45 kg/m2) groups due to the small number of patients.

Considering that randomized clinical trials for ERAS protocols are difficult to be implemented due to the significant risk of bias, further research with well-designed multicenter studies that stratify patients according to different obesity classes would provide stronger evidence and practical experience on the management of this challenging group of patients. Additionally, further research on multimodal and active prehabilitation programs would provide data on how to minimize obesity-associated morbidity.

## Conclusions

In conclusion, our findings suggest that the use of ERAS protocols can be safely implemented in obese patients with gynecological cancer treated with an open (laparotomic) approach while minimizing but not eliminating the potential multivariate associated morbidity. Obesity is a major disincentive factor for maximal surgical effort in patients with gynecological malignancies. However, these high-risk patients can be managed effectively by experienced multidisciplinary teams using ERAS protocols.
